# Evolutionary dynamics and antigenic diversity of porcine epidemic diarrhea virus (PEDV) in China: phylogenetic and recombination analyses based on large-scale S gene sequences

**DOI:** 10.1186/s12917-025-04873-y

**Published:** 2025-07-02

**Authors:** Yongjie Fu, Yingchun Wang, Liuliu Dai, Bimei Cheng, Shuqi Xiao, Yupeng Yin

**Affiliations:** 1 College of Animal Science and Technology, Northwest A&F University, Yangling, Shaanxi China; 2 College of Veterinary Medicine, Northwest A&F University, Yangling, Shaanxi China; 3 State Key Laboratory for Animal Disease Control and Prevention, Lanzhou Veterinary Research Institute, Chinese Academy of Agricultural Sciences, Lanzhou, Gansu China

**Keywords:** PEDV, S gene, Recombination, N-Glycosylation site, Evolution

## Abstract

Porcine epidemic diarrhea virus (PEDV) is a significant pathogen severely threatening the global swine industry. Due to its high variability, traditional vaccines have limited effectiveness in prevention and control. In this study, we conducted a systematic analysis of the molecular characteristics and evolutionary dynamics of 1109 PEDV strains circulating in China, focusing on their S gene sequences. Phylogenetic analysis revealed that Chinese PEDV strains could be classified into two major groups: GI (classic) and GII (variant), which were further divided into six subtypes: GIa, GIb, S-INDEL, GIIa, GIIb, and GIIc. Among them, GIIa accounted for 35.62%, GIIb for 25.70%, and GIIc for 26.06% of the total subtypes. Geographical distribution data showed that Guangdong, Sichuan, and Henan were the provinces with the highest incidences of PEDV. Analysis of amino acid mutations in the S protein suggested that GII strains might sustain transmission under immune pressure via a dual strategy of "sialic acid high affinity + immune escape". Recombination analysis indicated that the D0 structural domain, identified as a recombination hotspot, likely drives PEDV cross-species transmission and immune evasion. Glycosylation pattern analysis further demonstrated that GII strains exhibited unique glycosylation patterns at N62 and N118, potentially related to changes in immunogenicity. This study reports amino acid mutations, recombination events, and glycosylation site variation characteristics of Chinese PEDV strains. These findings provide a crucial foundation for elucidating the evolutionary mechanisms of PEDV, optimizing vaccine design, and formulating region-specific prevention and control strategies.

**Supplementary Information:**

The online version contains supplementary material available at 10.1186/s12917-025-04873-y.

## Introduction

Since 2010, highly pathogenic variant strains of porcine epidemic diarrhea virus (PEDV) have been increasingly spreading across the global pig industry, inflicting substantial economic losses. This disease is marked by acute watery diarrhea, vomiting, and dehydration. Notably, the mortality rate among newborn piglets is extremely high, frequently reaching 100%. As such, there is an urgent imperative for in-depth investigations into the disease, its causative pathogen, and the prompt development of effective vaccines tailored to prevalent strains [1]. PEDV is an enveloped, positive-sense single-stranded RNA virus. It belongs to the family Coronaviridae, genus Coronavirus, with an RNA genome of about 28 kb. This genome contains seven open reading frames that encode four structural proteins: the spike (S) protein, the membrane (M) protein, the envelope (E) protein, and the nucleocapsid (N) protein. Additionally, it encodes sixteen non-structural proteins (nsp1-nsp16) and an accessory protein, ORF3 [2]. PEDV was initially detected in growing and fattening pigs in the UK in 1971 [3] and in Belgium in 1978 [4]. In 2013, it was first reported in the USA [5], spreading rapidly within months and inflicting heavy losses [6]. PEDV was first isolated in China in 1984, and although it began to circulate in Chinese herds, no large-scale outbreaks occurred initially [7]. However, at the end of 2010, an outbreak emerged in several southern Chinese pig-producing provinces. Subsequently, the disease disseminated to other parts of China, resulting in significant economic losses [8]. Currently, PEDV infections are prevalent in major pig-producing countries across Asia, Europe, and North America [9].

Since the emergence of the PEDV, changes in its transmission process have given rise to numerous strains with varying virulence and infectivity. Through analyzing the evolution and genotypes of PEDV, the virus is typically classified into classical strains (GI) and mutant strains (GII). The mutant strains (GII) can be further subdivided into non-S-INDELs and S-INDELs [10]. Classical strains predominantly include early PEDV strains detected in Europe and Belgium, such as the CV777 strain, along with some common strains and most cell culture-adapted mutant strains (attenuated CV777 and DR13) obtained through successive in vitro passages [11]. Although classical strains have been identified in most regions of Europe and Asia, their relatively weak virulence mainly leads to sporadic outbreaks [9]. After 2010, a large-scale PEDV epidemic occurred in China. However, the vaccines against the classic strains had insufficient efficacy against the epidemic strains, indicating the emergence of mutant PEDV strains with high virulence and high mortality rate for piglets [12]. In 2011, a novel PEDV mutant strain was discovered in China [8]. Subsequently, in 2014, another PEDV mutant strain, OH851, was identified in the United States [13]. Genome sequencing and analysis of PEDV strains isolated in North America showed that the emerging PEDV mutant strains had insertions and deletions in the S gene. This genetic change led to a decrease in the virulence and pathogenicity of these new mutant PEDV strains [14]. Consequently, they were named"S-INDEL PEDV"to differentiate them from"non-S-INDEL PEDV". Studies indicate that S-INDEL PEDV may originate from recombination events between classical and mutant strains [15, 16].

As of 2010, with the extensive application of vaccines based on the CV777 strain, classical PEDV strains had largely vanished. Nevertheless, following 2010, China witnessed a large-scale epidemic of GII PEDV, during which the mortality rate of piglets exceeded 90%. Vaccines designed against classical strains proved insufficiently effective against the epidemic-causing strains, indicating that viral evolution might enable the virus to evade host immunity through antigenic drift or recombination. However, the precise underlying mechanisms remained unclear. In this study, we investigated the genetic evolution of the PEDV S gene in China, concentrating on amino acid mutation analysis, recombination analysis, and N-glycosylation site prediction analysis. Our research uncovered three major evolutionary advantages of the GII type. These findings deepen our comprehension of the epidemiology of PEDV in China and offer an analytical foundation for formulating effective strategies to prevent and control PEDV.

## Method

### Data set compilation and initial sequence comparison

A total of 1109 PEDV S genes were retrieved from the GenBank database in NCBI for sequence analysis. All sequences were aligned by the MAFFT v7 online version with default parameters and trimmed in Geneious Prime v2024.0.5 software [17]. The log in number, strain name, year of collection, and geographic origin information of the sequences were recorded in the Supplementary Table.

To determine the polymorphism patterns of S protein mutations in each sub-spectrum, multiple sequence alignment and homology analysis were performed using the Clustal Omega method using Geneious Prime software.

### Phylogenetic and evolutionary dynamics analysis

Genotyping of Chinese PEDV strains based on S protein gene sequence analysis Phylogeny-based genotyping of 1109 PEDV strains from China was performed. The phylogenetic tree was constructed by the neighbor-joining method through MEGA v12 software, using the p-distance model, and validated by 1000 bootstrap replicates. Subsequently, the phylogenetic trees were annotated using the Interactive Tree of Life (iTOL) software (https://itol.embl.de/).

The S-gene-based Bayesian time-scale tree was generated by the Markov Chain Monte Carlo (MCMC) method [18] in BEAST v1.10.4 (using the Uncorrelated relaxed clock model), Bayesian Skyline model. Convergence of log files was evaluated in Tracer v1.7.2 and Bayesian Skyline Plots (BSPs) were constructed to estimate effective population sizes for different subtypes [19, 20]. After discarding the first 10% of aging, the maximum evolutionary branching credibility (MCC) tree for each run was summarized using TreeAnnotator v1.10.4. The apical genetic distances of the sequences were validated by regression using TempEst v1.5.3 [21].

### Recombination detection

To identify possible recombination events, the viral dataset was screened for recombination signals using seven algorithms in the Recombination Detection Program 5 (RDP5), including RDP, GENECONV, Bootscan, MaxChi, Chimera, SiScan, and 3seq. Only recombination events validated by at least six of these methods were determined to be significant, with an acceptable critical p-value of less than 0.01.

### Prediction of S protein N-glycosylation sites

To explore the N-glycosylation pattern of Chinese PEDV strains, S protein sequences were submitted to the NetNGlyc web server (https://services.healthtech.dtu.dk/services/NetNGlyc-1.0/) for analysis. A default threshold of 0.5 was used to predict potential highly specific N-glycosylation sites.

## Results

### Phylogenetic analysis based on S gene sequences and geographic distribution of PEDV in China

**Fig. 1:**
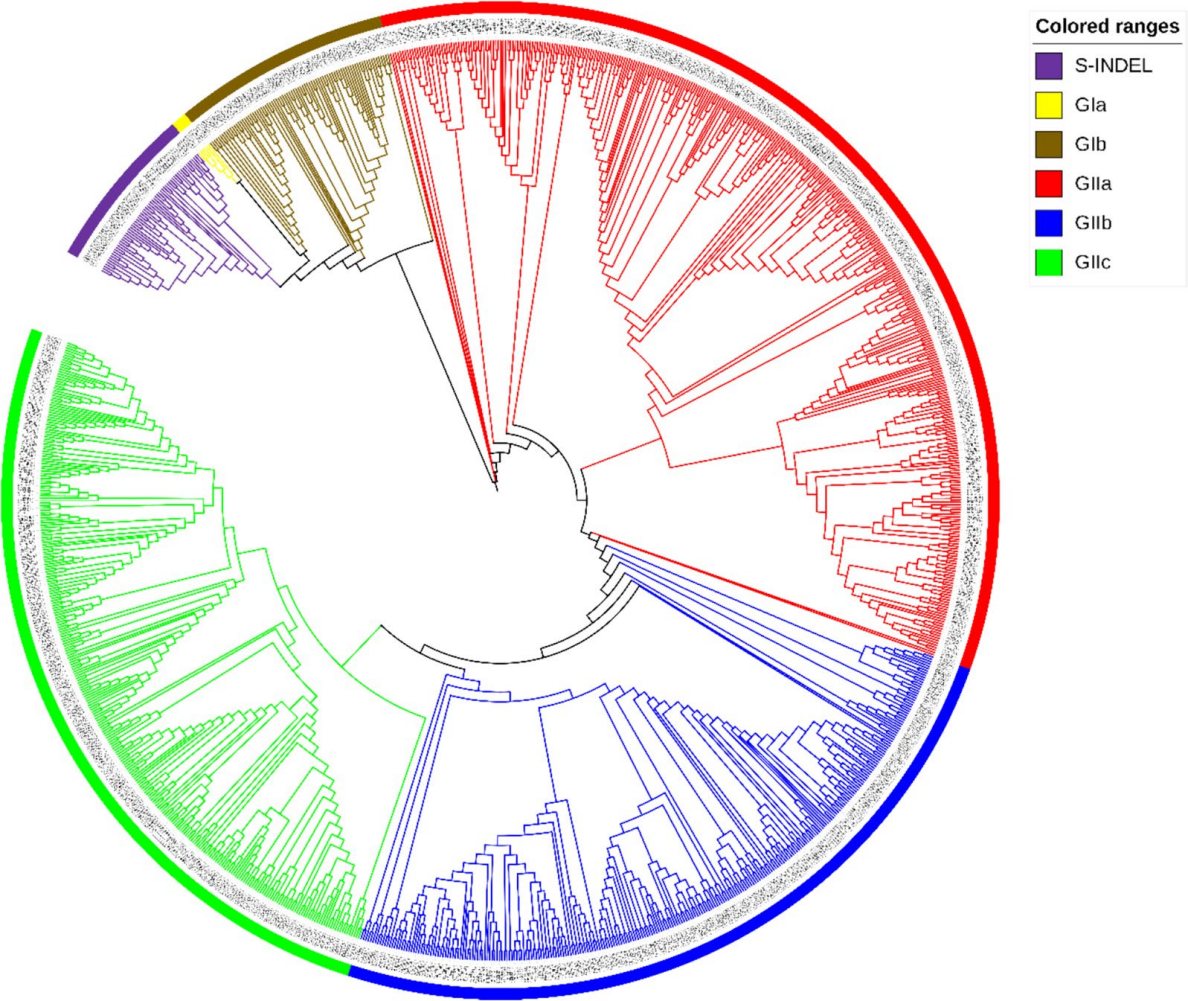
Phylogenetic analysis of S gene sequences of 1099 PEDV strains retrieved in this study. The tree was constructed using the neighbor-joining method in MEGA software. The branching colors of the different gene clusters are as follows: GIa (yellow), GIb (brown), S-INDEL (purple), GIIa (red), GIIb (blue) and GIIc (green)

**Fig. 2:**
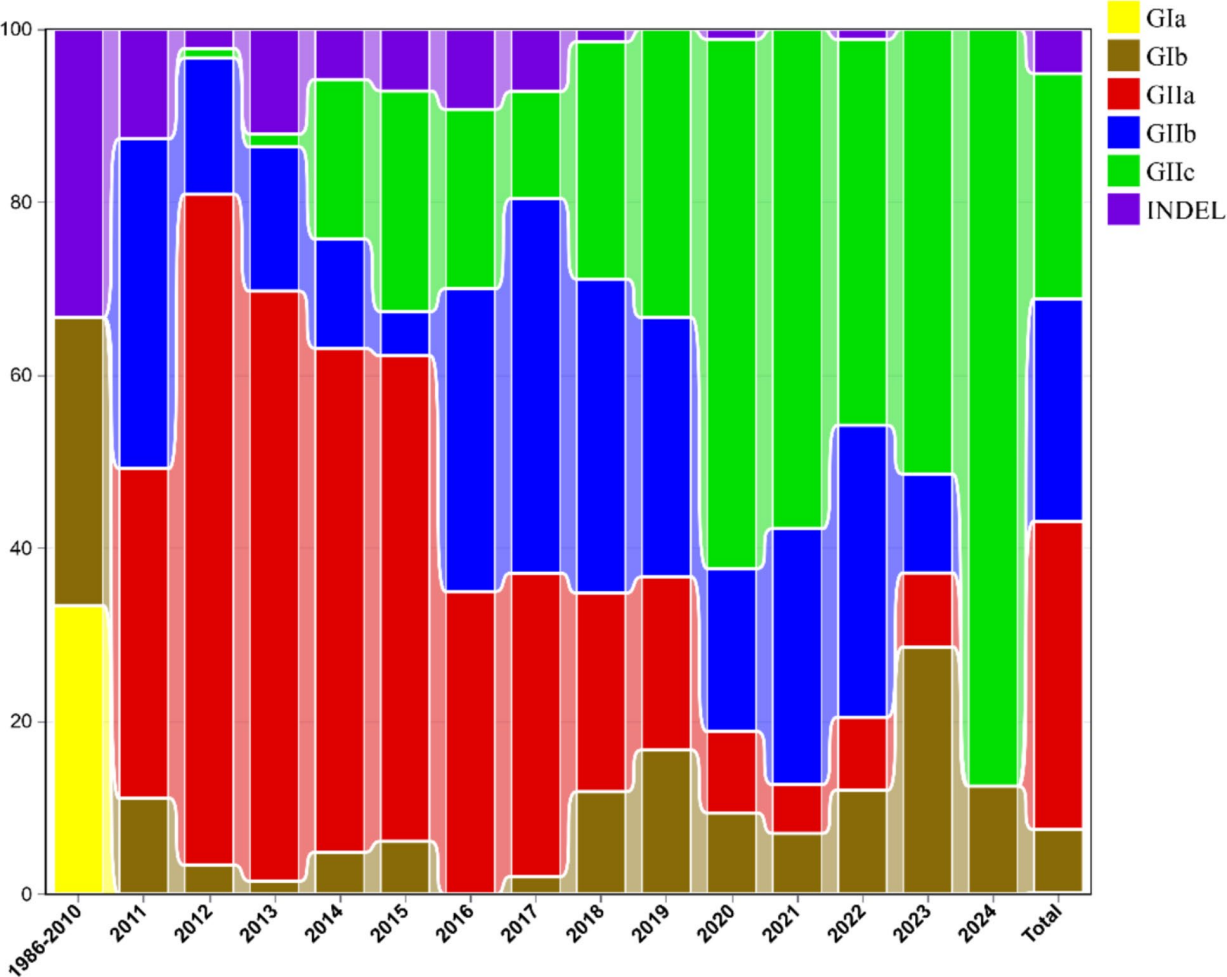
Relative frequency of different virus subtypes per year

**Fig. 3:**
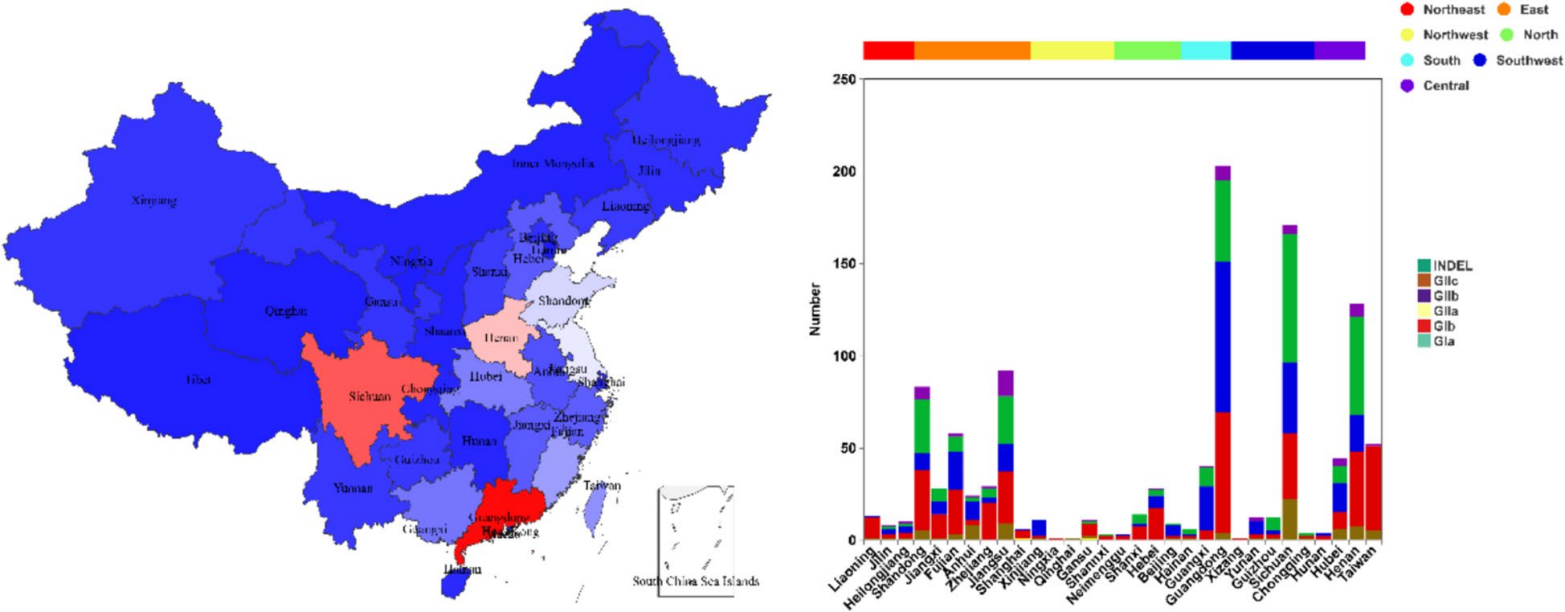
Geographic distribution of PEDV in different regions of China. Colors represent provincial isolate counts. Provinces are divided into seven regions based on proximity—North, Northeast, East, Central, South, Southwest and Northwest

To explore the genetic background of Chinese PEDV strains, a phylogenetic tree was constructed using MEGA software. This tree was based on the S gene sequences of 1109 Chinese PEDV strains and reference strains (Reference strain information is in the Supplementary Table) retrieved from GenBank. As shown in Fig. 1, Chinese PEDV strains were classified into two major groups: the GI-type (classic) and the GII-type (variant). These groups were further divided into six subtypes: GIa, GIb, S-INDEL, GIIa, GIIb, and GIIc. Specifically, 81 strains (7.30%) belonged to the GIb subtype, 57 strains (5.14%) to the S-INDEL subtype, 395 strains (35.62%) to the GIIa subtype, 285 strains (25.70%) to the GIIb subtype, and 289 strains (26.06%) to the GIIc subtype. As shown in Fig. 2, the results demonstrated that since 2010, GII-type PEDV strains have been highly prevalent in China, with the majority of the isolated strains belonging to this type. The proportion of GIIa subtype strains gradually declined after 2014, while GIIc subtype strains emerged and their numbers increased steadily. In contrast, the number of GIIb subtype strains remained relatively stable, and both GIIb and GIIc subtype strains have been the predominant strains isolated in recent years. As shown in Fig. 3, analysis of the geographical distribution of PEDV strains across China revealed that, in descending order, Guangdong, Sichuan, and Henan provinces had the highest prevalence of PEDV.

### Homology analysis

As shown in Table 1, the results indicated that when compared to the PEDV strain CV777 (AF353511.1), GIb-type strains displayed an average sequence identity of 95.4% (nucleotide, nt) and 95.0% (amino acid, aa) in the S gene. In contrast to the attenuated PEDV strain CV777 (KT323979.1), GIb-type strains showed an average sequence identity of 96.1% (nt) and 95.6% (aa). When compared with the highly virulent Chinese PEDV strain AJ1102 (JX188454.1), GIb-type strains exhibited an average sequence identity of 94.1% (nt) and 94.0% (aa) in the S gene. Notably, GIIa, GIIb, and GIIc subtype strains all demonstrated higher sequence similarity to the highly virulent Chinese PEDV strain AJ1102 and lower sequence similarity to the attenuated PEDV strain CV777, as opposed to GIb-type strains.

**Table 1:** Homology of Chinese PEDV S gene sequences with classical strain CV777 (AF353511.1), attenuated strain CV777 (KT323979.1) and variant strain AJ1102 (JX188454.1)

PEDV strains compared (GenBank accession no.)	NT/AA level	Identity
Compare ***GIb*** with CV777 strain (AF353511.1)	NT	93.4–96.9% ***(95.4%)***
	AA	92.1–96.3% ***(95.0%)***
Compare ***GIIa*** with CV777 strain (AF353511.1)	NT	91.5–97.2% ***(92.4%)***
	AA	90.7–96.6% ***(92.0%)***
Compare ***GIIb*** with CV777 strain (AF353511.1)	NT	92.3–94.2% ***(93.3%)***
	AA	90.3–93.7% ***(92.8%)***
Compare ***GIIc*** with CV777 strain (AF353511.1)	NT	92.1–93.4% ***(93.1%)***
	AA	91.4–93.4% ***(93.0%)***
Compare S-INDEL with CV777 strain (AF353511.1)	NT	93.6–96.1% (95.6%)
	AA	93.1–96.5% (95.9%)
Compare ***GIb*** with the CV777 attenuated strain (KT323979.1)	NT	93.0–99.9% ***(96.1%)***
	AA	91.5–99.8% ***(95.6%)***
Compare ***GIIa*** with the CV777 attenuated strain (KT323979.1)	NT	91.3–96.4% ***(92.2%)***
	AA	89.9–95.2% ***(91.3%)***
Compare ***GIIb*** with the CV777 attenuated strain (KT323979.1)	NT	91.9–93.5% ***(93.0%)***
	AA	89.6–92.8% ***(92.1%)***
Compare ***GIIc*** with the CV777 attenuated strain (KT323979.1)	NT	91.6–93.1% ***(92.7%)***
	AA	90.9–92.9% ***(92.3%)***
Compare S-INDEL with the CV777 attenuated strain (KT323979.1)	NT	93.3–96.1% (95.6%)
	AA	92.5–96.1% (95.6%)
Compare ***GIb*** with the AJ1102 strain (JX188454.1)	NT	90.4–96.2% ***(94.1%)***
	AA	89.7–96.0% ***(94.0%)***
Compare ***GIIa*** with the AJ1102 strain (JX188454.1)	NT	92.6–98.8% ***(97.4%)***
	AA	92.2–99.1% ***(97.7%)***
Compare ***GIIb*** with the AJ1102 strain (JX188454.1)	NT	96.7–100% ***(98.0%)***
	AA	95.6–100% ***(98.3%)***
Compare ***GIIc*** with the AJ1102 strain (JX188454.1)	NT	96.4–97.7% ***(97.4%)***
	AA	96.2–98.5% ***(98.0%)***
Compare S-INDEL with the AJ1102 strain (JX188454.1)	NT	94.1–97.5% (94.5%)
	AA	93.4–98.1% (94.6%)

### Comparison based on S protein amino acid sequence

The amino acid (aa) sequences of the S proteins of the typed strains were compared with those of reference strains to analyze the sequence variation of the typed strains. Mutations in the coronavirus S gene can impact both the immunogenicity of the S protein and its binding efficiency to the receptor. Previous research has pinpointed several neutralizing epitopes, namely COE (aa 499—638), SS2 (aa 748—755), SS6 (aa 764—771), and 2C10 (aa 1368—1374) [22, 23].

**Fig. 4:**
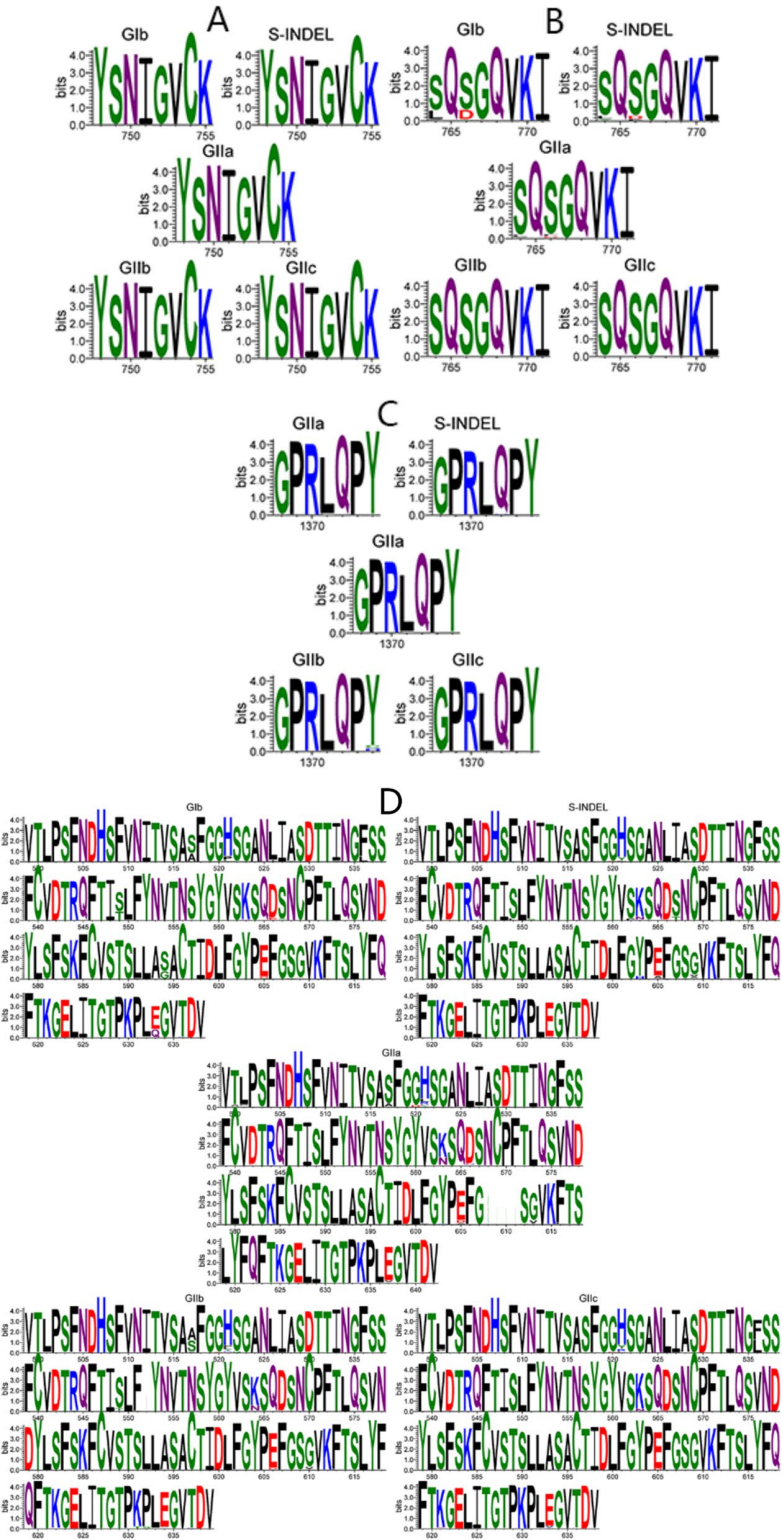
Amino acid mutation analysis of neutralization epitopes SS2 (**A**), SS6 (**B**), 2CIO (**C**), and COE (**D**) in Chinese PEDV S protein

As shown in Fig. 4, when comparing the neutralization epitope regions of the PEDV S protein with those of strain CV777, notable amino acid mutations were observed among different virus subtypes. In the SS6 neutralization epitope, most subtyped strains harbored the Y766S mutation, while a few GIb strains carried the S764L and Y766D mutations. Regarding the COE neutralization epitope, all subtype strains contained the L521H, S523G, V527I, T549S, G594S, A605E, L612F, and I635V mutations. Moreover, the majority of strains from the GIb, S—INDEL, GIIa, and GIIc subtypes also had the A517S mutation, although this mutation was present in only a small number of GIIa strains. Additionally, a portion of GIb strains showed L521H, S523G, V527I, A605E, L612F, E633Q, and I635V mutations within the COE neutralization epitope, and GIIa strains featured an insertion of filamentous proteins between sites 608—609. In contrast, the SS2 and 2C10 neutralization epitopes remained conserved across all subtype strains.

As shown in Table 2, when comparing other regions of the PEDV S protein with strain CV777, the GII strains showed more amino acid mutations than the GI strains. These mutations included Q27S, T29N, S55I, M56G, N57E, S58N, S60T, G64A, T65G, G66Q, I67H, E68P, Y80H, D82K(R), Q85H, D126S, N127I, D155E, I159S, A174S, L182F, R192K, R197G, S198G, T206E, Y223S, E225Q. Besides, there was an NQGV insertion at loci 57–58. In GIIa and GIIc subtype strains, there was an N insertion at loci 134–135, while GIIb subtype strains had an N insertion at loci 135–136.

**Table 2:** Analysis of amino acid mutations in Chinese PEDV S proteins and outside of epitopes

PEDV Subtypes	Amino Acid Mutations								
GIb	R2K	I5T	L10F	P15S	S28L	I67L	T113I	N114G	I116V
	R2T	S3P	L10F	P15L	T30I	A70D		MN114S153L	R154Q
S-INDEL	R2K	L10F	P15S	T30I	I67L	S83A	N114H	R154Q	D158N
GIIa	R2K	I5T	L10F	P15S	Q27S	S28A	T29N	S55I	M56G
GIIb	R2K	I5T	L10F	P15S	Q27S	S28A	T29N	S55I	M56G
GIIc	R2K	I5T	L10F	P15S	Q27S	S28A	T29N	S55I	M56G
GIb	T133S	V134S	D136G	T139S	Y152H	R154Q	D158N	V161I	R196K
	D158N	L182I	R196K	T232S	L282W	H298Q	V309A	D310Q	S324F
S-INDEL	R196K	T232S	H298Q	V309A	D310Q	I356T	E365Q	S454A	I667F
GIIa	N57E	57-58NQGV	S58N	S60T	G64A	T65G	G66Q	I67H	E68P
GIIb	N57E	57-58NQGV	S58N	S60T	G64A	T65G	G66Q	I67H	E68P
GIIc	N57E	57-58NQGV	S58N	S60T	G64A	T65G	G66Q	I67H	E68P
GIb	T232I	D242E	S243P	L266V	H298Q	M300I	D310Q	L338F	D351N
	L354A	A358T	A363V	N378K	R393K	I438V	S454A	A474S	I667F
S-INDEL	N707D	N719S	N724S	A877V	V893F	A959V	I963F	A965S	S1044A
GIIa	L78V	Y80H	D82K	S83G	Q85H	I116T	D126S	N127I	V134A
GIIb		Y80H	D82R	S83G	Q85H	I116T	D126S	N127I	V134A
GIIc		Y80H	D82R	S83G	Q85H	I116T	D126S	N127I	V134A
GIb	I356T	E365Q	L413I	S454A	I667F	N707D	N719S	N724S	A877V
	N707D	T774M	A806V	A877V	R891G	V893F	I963L	Y973H	K1023N
S-INDEL	N1047T	N1162D	L1164I	G1173D	T1193N	Y1194H	S1232R	R1298Q	N1302Y
GIIa	134-135N	Y152H	R154S	D155E	D158H	I159S	A174S	H179Y	L182F
GIIb	135-136N	Y152H	R154S	D155E	D158H	I159S	A174S		L182F
GIIc	134-135D	Y152H	R154S	D155E	D158H	I159S	A174S		L182F
GIb	V893F	A959V	I963F	A965S	L995M	S1044A	N1047T	N1162D	L1164I
	V1051I	L1164I	A1167D	E1174D	R1260I	N1302Y	V1332F		
S-INDEL	V1332F	G1359C	A1376V						
GIIa	R192K	R196S	R197G	S198G	T206E	Y223S	E225Q		T232I
GIIb	R192K	R196S	R197G	S198G	T206E	Y223S	E225Q		T232I
GIIc	R192K	R196S	R197G	S198G	T206E	Y223S	E225Q	P226L	T232I
GIb	G1173D	T1193N	Y1194H	S1232R	R1298Q	N1302Y	V1332F	G1359C	A1376V
S-INDEL									
GIIa	D242E	S243P	L266V		H298Q	M300I	D310Q	L342F	I356T
GIIb	D242E	S243P	L266V	I284M	H298Q	M300I	D310Q	L342F	I356T
GIIc	D242E	S243P	L266V	I284M	H298Q	M300I	D310Q		I356T
GIb									
S-INDEL									
GIIa	E365Q	S454A			I667F	N707D	N719S	N724S	
GIIb	E365Q	S454A	R487T	I496T	I667F	N707D	N719S	N724S	V843A
GIIc	E365Q	S454A			I667F	N707D	N719S	N724S	
GIb									
S-INDEL									
GIIa	A877V	V893F	A959V	I963F	A965S	S1044A	N1047T	N1162D	L1164I
GIIb	A877V	V893F	A959V	I963F		S1044A	N1047T		L1164I
GIIc	A877V	V893F	A959V	I963F	A965S	S1044A	N1047T	N1162D	L1164I
GIb									
S-INDEL									
GIIa	G1173D	T1193N				Y1194H	S1232R		
GIIb	G1173D	T1193D	Y1194 deletion	F1207Y	S1215G		S1232R	D1237E	P1265S
GIIc	G1173D	T1193N				Y1194H	S1232R		
GIb									
S-INDEL									
GIIa	R1298Q	N1302Y	V1332F	G1359C	A1376V				
GIIb	R1298Q	N1302Y	V1332F						
GIIc	R1298Q	N1302Y	V1332F	G1359C	A1376V				

### Analysis of evolutionary rates and population dynamics

**Fig. 5:**
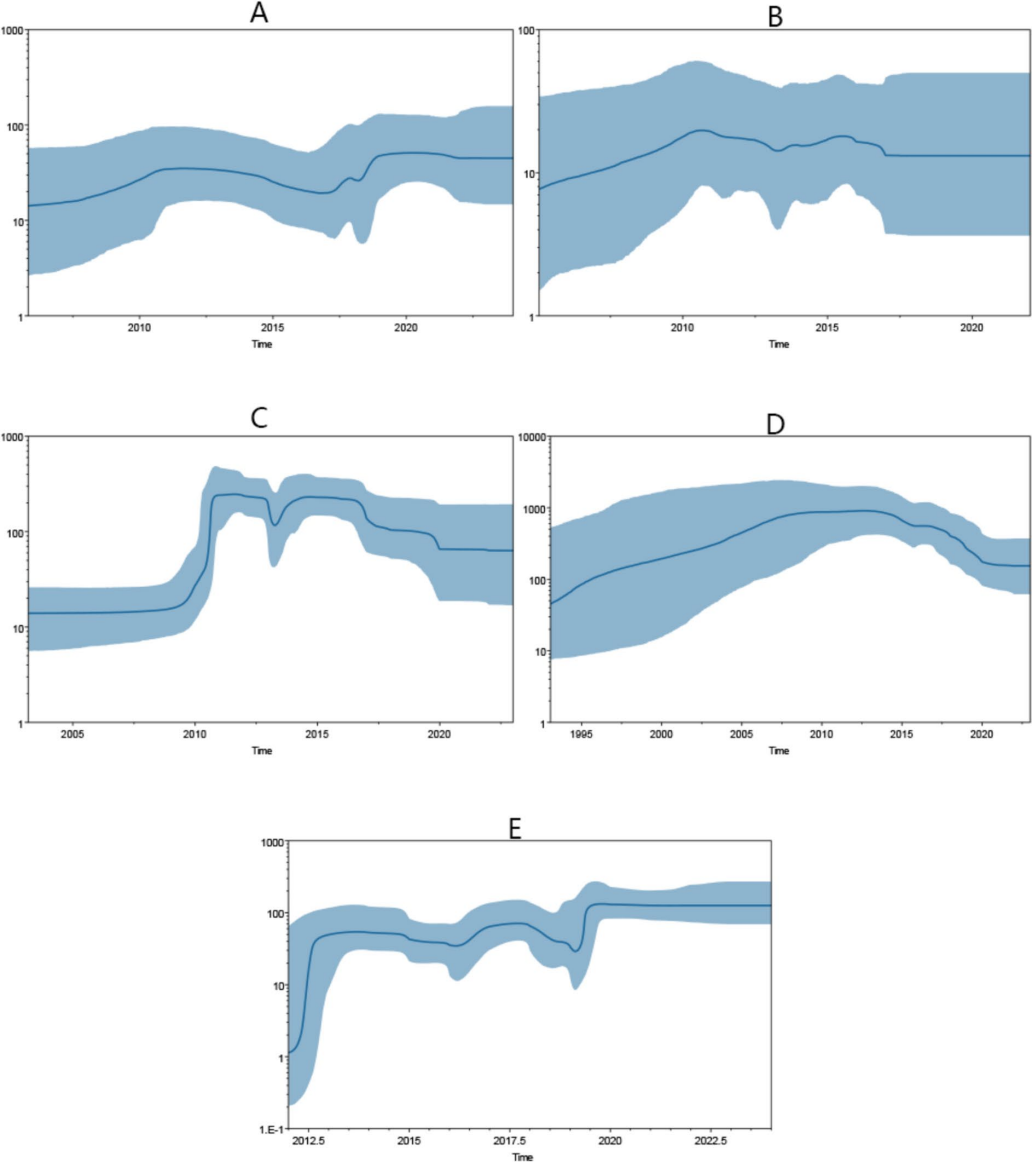
Effective population sizes (Ne) for PEDV GIb (**A**), S-INDEL (**B**), GIIa (**C**), GIIb (**D**), and GIIc (**E**) subtypes, with the blue line in the middle denoting the median estimate, and the shaded gray area denoting the 95% Confidence Interval (CI)

The analysis of the effective population size differences among PEDV subtypes, using the Bayesian skyline aggregation model, uncovered distinct fluctuating patterns of relative genetic diversity. As shown in Fig. 5(A), for the GIb subtype, its population size initially increased at a slow pace, started to decline around 2010, and then began to grow again and gradually stabilized around 2017. As shown in Fig. 5(B), the INDEL subtype first underwent a continuous and slow expansion, reaching its peak in 2010, after which its population size gradually declined with fluctuations until it plateaued in 2017. As shown in Fig. 5(C), The GIIa subtype's population size rapidly grew starting from 2010 and peaked in the same year. Subsequently, it began a slow decline and maintained a stable level after 2020. As shown in Fig. 5(D), The GIIb subtype initially expanded relatively rapidly and peaked in 2013, following which its population size gradually decreased. As shown in Fig. 5(E), for the GIIc subtype, its population size started to expand rapidly around 2012, and then stabilized. It began to grow again in 2019 and has since remained relatively stable.

Moreover, as shown in Table 3, the estimated mean evolutionary rates for different PEDV subtypes are as follows: The GIb subtype strains have a mean evolutionary rate of 1.44 × 10⁻^3^ (with a 95% highest posterior density (HPD) interval of 9.93 × 10⁻^4^—1.85 × 10⁻^3^); the INDEL subtype strains have an estimated mean evolutionary rate of 1.89 × 10⁻^3^ (95% HPD interval: 1.36 × 10⁻^3^—2.45 × 10⁻^3^); the GIIa subtype strains show a mean evolutionary rate of 1.92 × 10⁻3 (95% HPD interval: 1.70 × 10⁻^3^—2.1 × 10⁻^3^); the GIIb subtype strains have an estimated mean evolutionary rate of 6.76 × 10⁻^4^ (95% HPD interval: 3.78 × 10⁻^4^—1.03 × 10⁻^3^); and the GIIc subtype strains have a mean evolutionary rate of 1.41 × 10⁻^3^ (95% HPD interval: 1.22 × 10⁻^3^—1.59 × 10⁻^3^). The estimated divergence times for PEDV subtypes are: 1995.1 (95% HPD interval: 1980.9—2005.8) for the GIb subtype; 1990.6 (95% HPD interval: 1984.0—1996.3) for the INDEL subtype; 1994.4 (95% HPD interval: 1983.0—2003.2) for the GIIa subtype; 1969.7 (95% HPD interval: 1941.4—1993.0) for the GIIb subtype; and 2010.6 (95% HPD interval: 2008.6—2012.0) for the GIIc subtype.

**Table 3:** Mean evolutionary rate and divergence time of PEDV subtype strains

PEDV Subtypes	Mean Evolutionary Rate	Divergence Time
GIb	1.44 × 10⁻^3^ (95% HPD interval: 9.93 × 10⁻^4^—1.85 × 10⁻^3^)	1995.1 (95% HPD interval: 1980.9—2005.8)
S-INDEL	1.89 × 10⁻^3^ (95% HPD interval: 1.36 × 10⁻^3^—2.45 × 10⁻^3^)	1990.6 (95% HPD interval: 1984.0—1996.3)
GIIa	1.92 × 10⁻^3^ (95% HPD interval: 1.70 × 10⁻^3^—2.1 × 10⁻^3^)	1994.4 (95% HPD interval: 1983.0—2003.2)
GIIb	6.76 × 10⁻^4^ (95% HPD interval: 3.78 × 10⁻^4^—1.03 × 10⁻^3^)	1969.7 (95% HPD interval: 1941.4—1993.0)
GIIc	1.41 × 10⁻^3^ (95% HPD interval: 1.22 × 10⁻^3^—1.59 × 10⁻^3^)	2010.6 (95% HPD interval: 2008.6—2012.0)

### Recombination analysis of the PEDV S gene

Recombinant analysis was carried out using the RDP5 software on 282 strains. These strains were selected from 34 representative reference strains and the 1109 strains retrieved in the study, specifically those from 2020—2024. The analysis results indicated that among the 231 strains examined, 83 were recombinant. The recombinant strains were distributed as follows: 27 belonged to the GIb subtype, 1 to the S—INDEL subtype, 5 to the GIIa subtype, 37 to the GIIb subtype, and 14 to the GIIc subtype.

As shown in Table 4 (the end of this article), the results demonstrated that the majority of GIb recombinant strains were the result of recombination between GI and GII strains, with the recombination region encompassing Domain 0. For GIIb recombinant strains, most of them were recombinants of different GIIb strains (M609204.1, KU646831.1, and MZ161041.1), and the recombination regions predominantly included HR2, TM, and Domain 0. A small number of GIIb recombinant strains were formed by recombination between GIIb and S-INDEL strains, with the recombination region containing HR1. As for GIIc strains, some were recombinants of GIIb strains, and the recombination regions essentially covered FP and HR1 of S1 and S2. Additionally, some GIIc strains were recombinants of GIa and GIIb strains, and their recombination regions included Domain 0.

**Table 4:**
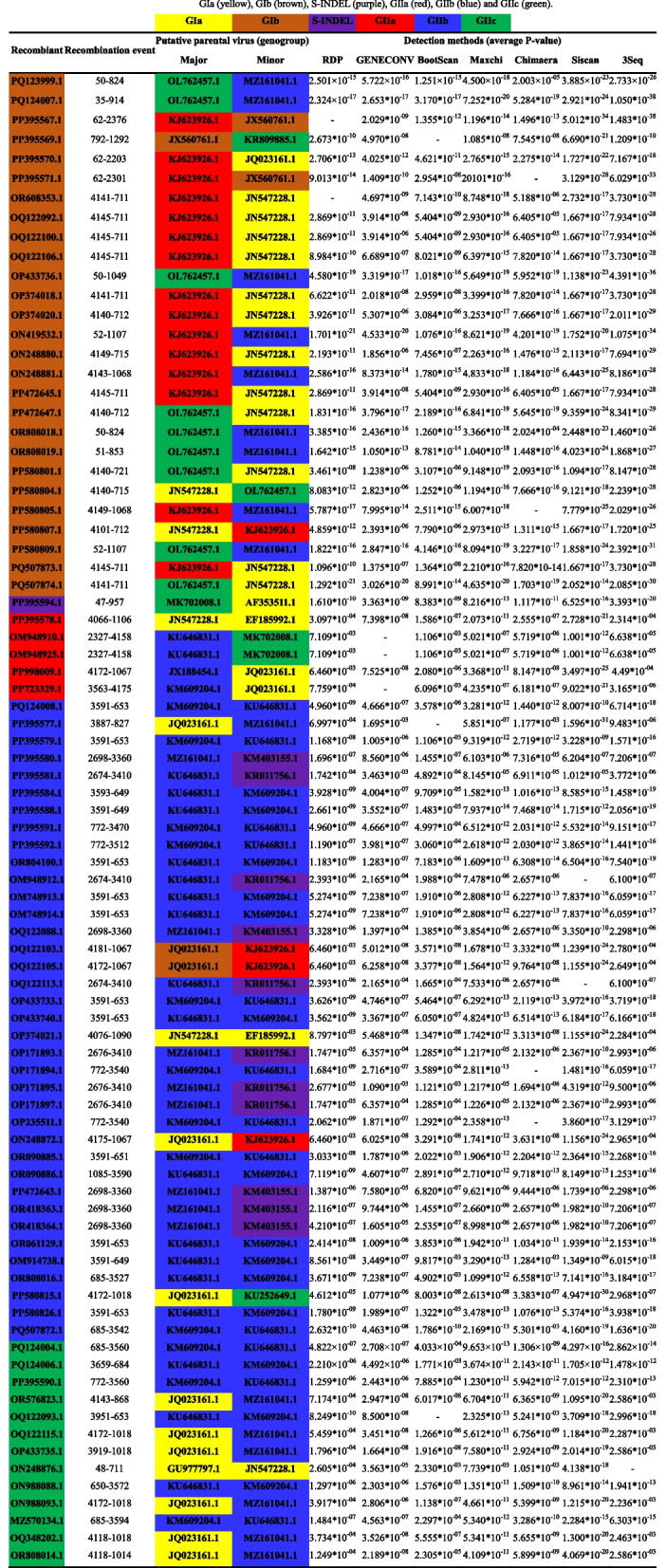
Information on recombination events identified in the S genes of 282 PEDV strains in China from 2020 to 2024

GIa (yellow), GIb (brown), S-INDEL (purple), GIIa (red), GIIb (blue) and GIIc (green)

### Highly specific glycosylation site pattern of PEDV S protein

**Fig. 6:**
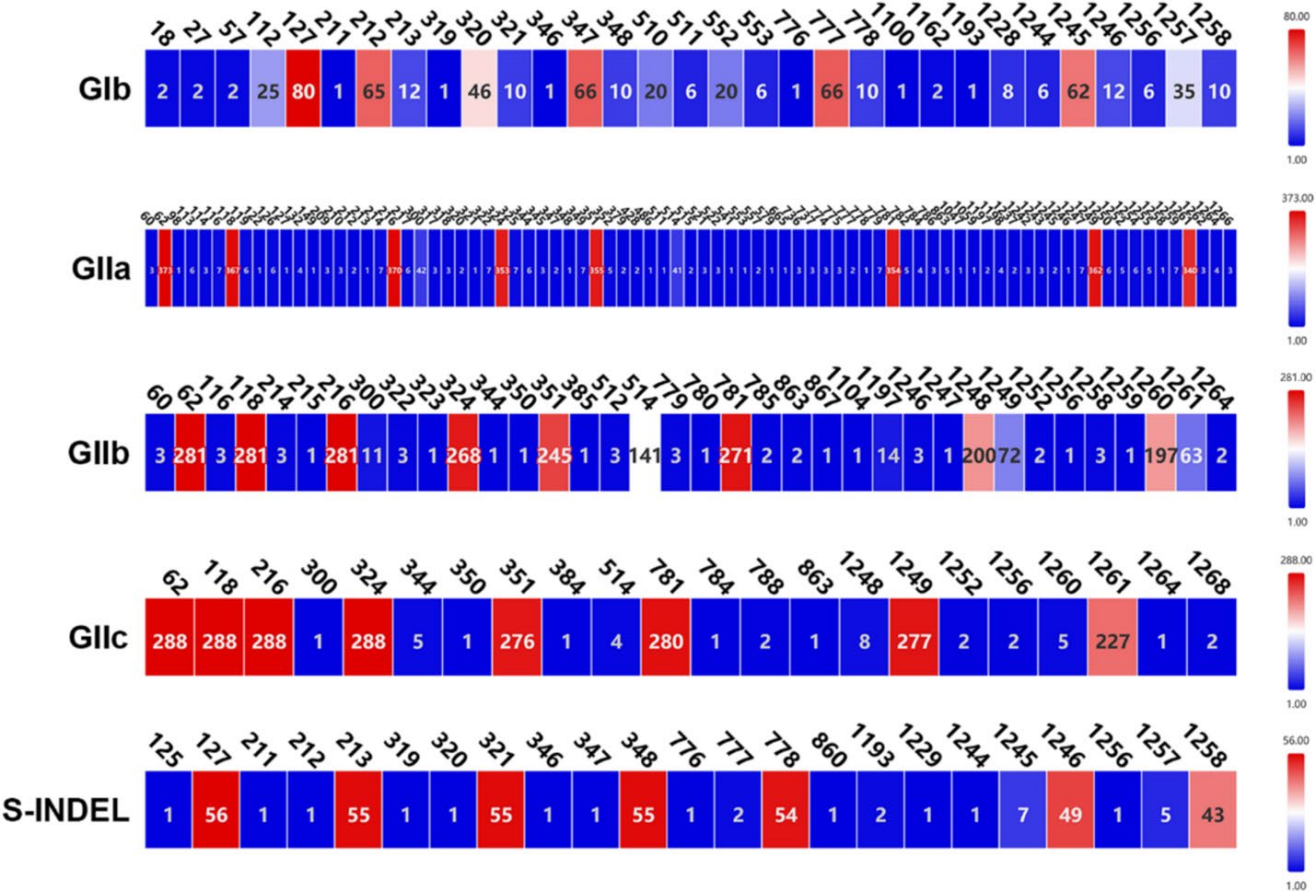
Changes in highly specific N-glycosylation sites in S proteins of different subtypes of strains compared to the CV777 vaccine strain. Different colors represent the number of strains that undergo N-glycosylation at this locus

N-glycosylation site information for each strain is in the Supplementary Table. Highly specific N-glycosylation site analyses among PEDV subtypes revealed variability in site occupancy patterns. Although glycosylation profiles were not strictly subtype-defining, some conserved patterns were observed. As shown in Fig. 6, the results showed that most GIb subtype strains exhibited N-glycosylation at sites 127, 212, 320, 347, 777, and 1245, whereas S-INDEL subtype strains exhibited N-glycosylation mainly at sites 127, 213, 321, 348, 778, 1246, and 1258. Also, GIIa and GIIc subtype strains showed N-glycosylation mainly at loci 62, 118, 216, 324, 351, 781, 1249, and 1261, whereas most GIIb subtype strains showed N-glycosylation at loci 62, 118, 216, 324, 351, 781, 1248, 1260. Most GIb strains lost N-glycosylation at loci 511, 553, 1258 compared to classical strain CV777 (127, 213, 321, 348, 511, 553, 778, 1246, 1258). Among the S-INDEL strains, most lost N-glycosylation at sites 511, 553. Also in GIIa, GIIb, and GIIc strains, most lost N-glycosylation at sites 127, 511, 553 and gained additional N-glycosylation at sites 62, 118.

## Discussion

Previous research has demonstrated that the Chinese strain of PEDV was initially isolated in 1984 using antibody detection and serum neutralization assays [7]. Until 2010, PEDV infections in China were sporadic, primarily because of the application of inactivated or attenuated vaccines [24]. However, the existing vaccines proved inadequate in combating variant PEDV strains, which caused substantial economic losses for the Chinese swine industry in 2010 [12]. As the world's largest live pig importer, China's large-scale piglet imports have fueled PEDV spread and diversification. Inter-provincial live pig trade also significantly drives domestic PEDV dissemination [25]. The high variability of PEDV field strains renders pandemics more complex and heterogeneous, posing a significant obstacle to effective immunization [26].

Phylogenetic analysis classified Chinese PEDV strains into six subtypes: GIa, GIb, S-INDEL, GIIa, GIIb, and GIIc. Of these, 81 strains (7.30%) were GIb, 57 (5.14%) were S-INDEL, 395 (35.62%) were GIIa, 285 (25.70%) were GIIb, and 289 (26.06%) were GIIc. Notably, GIIb and GIIc subtypes have been the predominant strains isolated in recent years. The provinces with the highest PEDV prevalence, in descending order, were Guangdong, Sichuan, and Henan. This finding aligns with He et al. [25], who demonstrated that inter-provincial live pig trade is a major driver of PEDV spread in China. Notably, Guangdong and Henan served as key virus transmission hubs, facilitating PEDV introduction to other regions. Furthermore, compared to the GIb subtype, GIIa, GIIb, and GIIc strains exhibited higher sequence similarity to the highly virulent Chinese PEDV strain AJ1102 and lower sequence similarity to the attenuated PEDV strain CV777. This observation partially elucidates why the conventional PEDV CV777 vaccine fails or shows limited efficacy against pigs infected with mutated PEDV strains.

When comparing the neutralizing epitope region of the PEDV S protein with that of the virulent strain CV777, the SS2 and 2CIO neutralizing epitopes were found to be conserved across all subtypes. This conservation suggests the potential for developing a universal subunit vaccine targeting these epitopes to overcome the antigenic disparities between existing vaccine strains and epidemic strains. However, additional research is required to confirm the high structural stability of these epitopes. In the other regions of the PEDV S protein, GII strains have acquired new amino acid mutations relative to GIb strains. These mutations include Q27S, T29N, S55I, M56G, N57E, S58N, S60T, G64A, T65G, G66Q, I67H, E68P, Y80H, D82K(R), Q85H, D126S, N127I, D155E, I159S, A174S, L182F, R192K, R197G, S198G, T206E, Y223S, and E225Q. Moreover, an NQGV insertion occurs at positions 57—58. These mutations and insertions predominantly occur within the Domain 0 region. The D0 structural domain mediates binding to sialic acid, a potential virulence factor for PEDV strains [27, 28]. This sialic acid-binding ability enables PEDV to accumulate on the surfaces of host cells, particularly intestinal epithelial cells, thereby enhancing the virus's specific infection of intestinal tissues [29, 30]. As a critical target of the host immune system, antibodies targeting the D0 domain can exert neutralizing effects through two mechanisms: blocking sialic acid binding or inducing conformational changes [31]. Mutations and insertions in GII strains may have optimized their binding conformation to sialic acid. The insertions could facilitate the formation of a more stable receptor-binding site, enhancing the affinity for sialic acid on intestinal epithelial cell surfaces. This provides a molecular basis for potential cross-species transmission. Domain 0 is a key target for neutralizing antibodies. Mutations in GII strains might enable them to evade neutralization by GI-specific antibodies through spatial site-blocking or epitope conformation changes [10, 32, 33]. Additionally, some of these mutations may abolish potential N-glycosylation sites, exposing cryptic epitopes and thus disrupting immunorecognition. Mutations in Domain 0 may increase the S protein's resistance to intestinal trypsin, extending the time window for virus particle infection in the intestine. Alternatively, they might enhance the virus's stability at low temperatures, improving its environmental adaptability. These Domain 0 mutations could also alter the conformation of the receptor-binding domain (RBD) of the S1 subunit via allosteric effects, facilitating the binding of the S1 subunit to porcine aminopeptidase N (pAPN) or enhancing binding to novel receptors. Consequently, the nationwide prevalence of GII strains is closely associated with the accumulation of these mutations. Their continuous transmission under immune pressure is accomplished through a dual strategy of "sialic acid high affinity + immune escape".

Besides the accumulation of locus mutations, homologous recombination is another prevalent pattern of genetic evolution in coronaviruses [34]. Genomic recombination has the potential to modify viral pathogenicity and transmissibility [32]. In this research, recombination analysis of the S genes of recent PEDV strains detected 83 potential recombination events. Among them, 27 were GIb strains, 1 was an S-INDEL strain, 5 were GIIa strains, 37 were GIIb strains, and 14 were GIIc strains. The findings demonstrated that the majority of the major parental strains of the recombinants were GII PEDVs, suggesting that GII subtype PEDVs continue to pose a threat to China's pig industry. Moreover, most GIb recombinant strains resulted from recombination between GI and GII strains, and the recombination region included Domain 0. The recombination of GIb recombinant strains in the D0 structural domain of the S1 subunit is, in essence, an evolutionary strategy adopted by viruses under natural selection pressure. Firstly, it involves immune escape mutations (such as insertions/deletions or key amino acid substitutions) to evade the immune responses triggered by conventional vaccines or natural antibodies against GI-type strains. Secondly, GI-type strains tend to maintain conserved sialic acid-binding sites, while GII-type strains may introduce mutations through recombination to enhance their adhesion to intestinal epithelial cells. Recombinant GIb strains may combine the advantages of both, thereby increasing infection efficiency in the intestinal environment and expanding their host range. Most GIIb recombinant strains are formed by the recombination of GIIb strains (M609204.1, KU646831.1, and MZ161041.1), and the majority of the recombinant regions include HR2, TM, and Domain 0. HR2 and TM are situated in the S2 subunit of the S protein and are both involved in the membrane fusion process between viruses and host cells. Variations in their sequences may impact the membrane fusion efficiency, thereby changing the virus's infectivity [35]. The recombination of the same GIIb subtype strain for fine-tuning optimization maintains a high degree of genomic conservation and circumvents the potential adaptation costs associated with cross-subtype recombination. Recombination through the HR2-TM-Domain region enhances infection efficiency (fine-tuning optimization of the whole process of adhesion → fusion → release), immune escape (remodeling of Domain 0 neutralizing epitopes + masking of HR2 non-neutralizing epitopes), and environmental adaptability (enhanced stability of the TM + cryoactivity of the HR2). The remaining few are recombinants of GIIb and S-INDEL strains, and their recombinant regions all contain HR1. As a key structural domain of the PEDV S protein, HR1 works in tandem with HR2 to mediate membrane fusion between the virus and host cells [35]. Through integrating the advantageous mutations of GIIb and S-INDEL, recombination may optimize the fusion efficiency, thereby enhancing the virus's infectivity towards host cells. GIIb strains are highly transmissible, whereas S-INDEL strains carry immune escape mutations (such as insertions or deletions in S-INDEL that change antigenicity). Recombination could generate viruses with even faster transmission rates and the ability to evade part of the immune response. Different strains may possess adaptive advantages in specific environments or host populations, and by recombining, they can further boost their adaptability within host populations. Furthermore, some recombinant strains seem to be novel ones formed by the recombination of domestic and foreign strains within China. Given that China is the world's leading live pig importer, it is postulated that the appearance of these new recombinant strains is linked to the international live pig trade [36]. Economic globalization and cross-country live pig trade not only heightens the likelihood of recombination among PEDV strains from various regions but also facilitate the large-scale transmission and dissemination of PEDV [36].

Viral N-glycosylation plays an important role in host cell attachment and release, glycan shielding, immune escape, and infection [37]. Increased N-glycosylation sites and changing glycosylation patterns provide key evidence for adaptive viral evolution in response to host humoral immunity [38]. In this study, distinct patterns of highly specific N-glycosylation were observed between GI and GII PEDV strains. The GIb and S-INDEL PEDV strains exhibited a similar N-glycosylation site pattern, showed close genetic relatedness to the CV777 strain, and lacked novel highly specific N-glycosylation sites compared with CV777. Conversely, the GIIa, GIIb, and GIIc strains shared a comparable N-glycosylation site pattern and presented new highly specific N-glycosylation sites (N62 and N118) relative to the CV777 strain. Notably, all these new sites were situated within the Domain 0 region. Therefore, the newly added glycosylation sites in GII strains likely significantly boost the infection efficiency and immune escape capacity of PEDV. This occurs through optimizing sialic acid-binding ability, which promotes virus attachment and broadens the host range; masking key antigenic epitopes for immune evasion; and enhancing environmental adaptability. This evolutionary trait elucidates the high pathogenicity of GII strains to neonatal piglets and their ability to evade conventional vaccines. Additionally, the variations in the N-glycosylation site patterns of GII strains are confined to the last two sites, probably as a result of viral evolution. Previous research has demonstrated that mutations in the N-glycosylation site of the PEDV S protein enhanced the immunogenicity of AH2012/12 [33]. It was hypothesized that the removal of the glycosylation site could expose the immunogenic epitopes of the S protein, thereby reducing the virus's capacity to evade the host immune response. Thus, the identification of the N-glycosylation sites in the S protein of Chinese PEDV strains may serve as a basis for devising strategies to modify these sites, aiming to develop vaccine candidates capable of eliciting high-titer neutralizing antibodies.

This study elucidated three primary evolutionary characteristics of Chinese PEDV strains: (1) ecological niche occupation of type GII: Type GII strains manifested mutations such as Q27S, T29N, S55I, M56G, N57E, S58N, S60T, G64A, T65G, G66Q, I67H, E68P, Y80H, D82K(R), Q85H, D126S, N127I, D155E, I159S, A174S, L182F, R192K, R197G, S198G, T206E, Y223S, E225Q in the D0 structural domain. Additionally, an insertion mutation at locus 57—58 (NQGV) was noted. These changes likely enabled the strains to adopt a “salivary acid high affinity + immune escape” dual strategy, allowing for continuous transmission under immune pressure. Moreover, the interprovincial live pig trade expedited their geographical dissemination [25]. This discovery aligns well with the recently reported expansion trend of PEDV strains in Southeast Asia [34]. (2) Recombination hotspot D0 structural domain: The recombination hotspot of the PEDV S gene resides in the D0 structural domain, indicating its potentially pivotal role in viral adaptive evolution. The β-folding conformation of the D0 structural domain directly engages in spatial interactions with the sialic acid glycan chains on the surface of host cells. Genetic variability within this region likely drives PEDV's cross-species transmission and immune escape by modifying binding affinity or receptor specificity. (3) Immunomodulatory role of glycosylation modifications: Glycosylation at N62 and N118 in GII strains reduces antibody recognition efficiency by masking neutralizing epitopes, which aligns with the findings of Ma et al. [31]. Subunit vaccines designed to target deglycosylated forms may potentially overcome the current immune evasion barriers.

In this study, by integrating multi-omics data and evolutionary characteristics, the following PEDV prevention and control strategies were put forward: (1) Vaccine Design: Develop a multivalent mRNA vaccine based on GII-type epitopes, such as deglycosylated N62 and N118. (2) Monitoring System: Establish an early-warning platform for PEDV evolution relying on real-time genome sequencing, with a focus on recombination hotspot regions. (3) Cross-species Risk Assessment: Analyze the potential infection risk of recombinant PEDV strains to non-swine hosts, including dogs and cats. In the future, further analysis of the functional mechanisms of glycosylation sites is required, along with an exploration of the potential applications of mRNA vaccines in PEDV prevention and control.

## Supplementary Information

Supplementary Material 1

## Data Availability

All data analyzed during this study are publicly available nucleotide sequences deposited in the GenBank database (https://www.ncbi.nlm.nih.gov/genbank/). The complete list of GenBank accession numbers for Porcine Epidemic Diarrhea Virus (PEDV) spike (S) gene sequences analyzed in this study is provided in Supplementary Table S1.
